# Isolated attosecond X-ray pulses from superradiant thomson scattering by a relativistic chirped electron mirror

**DOI:** 10.1038/s41598-022-24288-1

**Published:** 2022-11-17

**Authors:** B. H. Schaap, P. W. Smorenburg, O. J. Luiten

**Affiliations:** 1 Department of Applied Physics, University of Technology Eindhoven, 5600 MB Eindhoven, The Netherlands 513,; 2 ASML Netherlands B.V., 5500 AH Veldhoven, The Netherlands 324,

**Keywords:** X-rays, Physics, Attosecond science

## Abstract

Time-resolved investigation of electron dynamics relies on the generation of isolated attosecond pulses in the (soft) X-ray regime. Thomson scattering is a source of high energy radiation of increasing prevalence in modern labs, complementing large scale facilities like undulators and X-ray free electron lasers. We propose a scheme to generate isolated attosecond X-ray pulses based on Thomson scattering by colliding microbunched electrons on a chirped laser pulse. The electrons collectively act as a relativistic chirped mirror, which superradiantly reflects the laser pulse into a single localized beat. As such, this technique extends chirped pulse compression, developed for radar and applied in optics, to the X-ray regime. In this paper we theoretically show that, by using this approach, attosecond soft X-ray pulses with GW peak power can be generated from pC electron bunches at tens of MeV electron beam energy. While we propose the generation of few cycle X-ray pulses on a table-top system, the theory is universally scalable over the electromagnetic spectrum.

## Introduction

Atomic and molecular dynamics is governed by forces caused by electron motion, which changes at the attosecond time scale. For about two decades the main driver of attosecond science has been high harmonic generation from noble gasses^1^. High harmonics have enabled the imaging of electron wave-packets in motion of several atomic^2,3^ and molecular species^4–6^. However, the insufficient photon flux of isolated attosecond pulses from high harmonics, especially at high photon energy^7^, limits applications^8^. To increase the amount of photons in attosecond pulses for advanced applications, undulator based alternatives have been proposed^9–14^ and demonstrated^15,16^ over the years, yet at the cost of real estate.

Thomson scattering (TS) of relativistic electrons, also referred to as inverse Compton scattering, is an upcoming, lab-based X-ray source^17–22^. In a Thomson X-ray source, a relativistic electron bunch reflects high energy radiation of a laser pulse. Both the velocity of the electrons *v*, the angular frequency of the laser pulse $\omega _L$ determine the scattered angular frequency by the well known equation

1 $$\begin{aligned} \omega _1 = \omega _L\frac{1 + v/c \cos \theta _L}{1 - v/c \cos \theta }, \end{aligned}$$

where *c* the speed of light, $\theta _L$ the laser angle of incidence and $\theta$ the scattering angle as measured from the propagation axis of the electron beam. Thomson scattering could bridge the gap between the large scale undulator schemes and low flux HHG-based, and other lab sources. To extend TS sources into the attosecond realm, several schemes have been proposed that employ single cycle driver pulses^23,24^ and attosecond electron bunches^25,26^.

**Figure 1:**
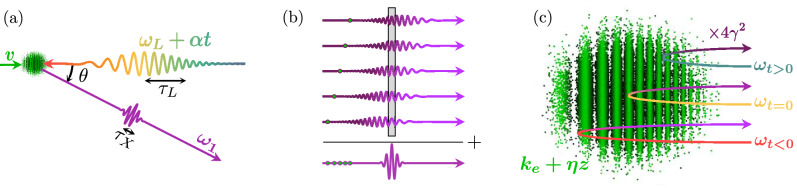
(**a**) Attosecond X-ray pulse (purple) generation by colliding microbunched electrons (green) on a chirped laser pulse (gradient). (**b**) Single electron radiation fields (purple gradient) of axially separated electrons (green) and the resulting superradiant beat (purple). The grey box illustrates the part that contributes to superradiance. (**c**) A relativistic chirped electron mirror has a frequency dependent depth at which superradiance occurs.

In this paper, we propose a scheme to generate isolated attosecond X-ray pulses via TS by colliding microbunched electrons on a chirped laser pulse as shown in Fig. 1a. The electrons are distributed in such a way that the frequency up-converted radiation pulses interfere constructively into a single localized beat, as illustrated schematically in Fig. 1b. The scheme is attractive since both the electron bunch and laser pulse can be of long temporal extent, making high power attosecond pulses possible, while mitigating detrimental space charge effects.

In essence, this scheme extends the chirped pulse compression technique employed in radar^27^ and optics^28^ to the X-ray regime, similar to proposed FEL-based techniques^12,13^. In chirped pulse compression, an amplified frequency modulated signal is sent through chromatic delay line so that the frequencies pile up (compress) in time to form a short pulse. In optics a customary delay line is a chirped mirror having a frequency dependent penetration depth. Our scheme relies on superradiant emission from microbunched electrons that, very similarly, act as a relativistic chirped electron mirror (CEM) with a frequency dependent depth at which superradiance occurs, as depicted schematically in Fig. 1c.

Superradiant emission, describing only the constructive interfering part of the reflected radiation, occurs when the phase difference of the radiation from spatially separated emitters is equal to an integer *n* of $2\pi$. For superradiant TS of radiation at frequency $\omega _1$, a density modulated electron beam should have a bunching frequency^29^

2 $$\begin{aligned} k_e = \frac{\omega _L}{n c}\frac{\cos \theta _L + \cos \theta }{1 - v/c \cos \theta }. \end{aligned}$$

However, in chirped pulse compression the laser frequency is not constant but varies with time. The use of a frequency chirped laser pulse has been proposed before to compensate for bandwidth broadening in incoherent TS^30–32^. Here, in contrast, we want to increase the bandwidth of the generated radiation by means of frequency chirp to attain short X-ray pulses. Therefore, the bunching frequency of the electron mirror should also vary.

For optimal chromatic delay, the periodic density modulation of the mirror should satisfy the following two conditions. First, the variation in bunching frequency should be large enough to support superradiant reflection of the full laser bandwidth. Second, the superradiant reflection of each spectral component should occur in a single beat, which can only hold true if the periodic density modulation of the electron mirror is tailored to the frequency chirp of the laser pulse. For instance, if the laser pulse has a linearly chirped frequency $\omega _L + \alpha t$, with $\alpha$ the chirp rate, then the bunching frequency $k_{e} + \eta z$ should also vary linearly along the length *z* of the mirror, where $\eta$ the spatial chirp rate of the density modulation. By correctly matching the chirp rate of the laser $\alpha$ to the mirror chirp rate $\eta$, it is possible to attain few cycle X-ray pulses or high peak power pulses.

Here, we will use analytical and numerical methods to demonstrate under which conditions isolated attosecond X-ray pulses are generated by chirped laser pulse compression and frequency up-conversion using a relativistic chirped electron mirror. The framework of classical electrodynamics will be applied to linear Thomson scattering of a chirped laser pulse, in order to predict the attosecond pulse length and peak power.

## Results

We consider chirped pulse compression and frequency upconversion of a linearly chirped laser pulse by a relativistic chirped mirror consisting of free electrons. Our calculations, see Methods, show that the rms pulse length of the superradiantly reflected X-ray pulse, resulting from a head-on interaction is given by

3 $$\begin{aligned} \tau _X = \left[ \tau _\mathrm{min}^2 + \tau _\mathrm{CEM}^2 + \tau _\perp ^2 + \tau _\mathrm{GDD}^2\right] ^{1/2}. \end{aligned}$$

As indicated by Eq. (3) the generated X-ray pulse length takes the form of a quadratic sum of several contributions. We will now discuss these contributions one by one. The first term $\tau _\mathrm{min} = (c - v \cos \theta )/((c+ v)\Omega _L)$ is the shortest attainable pulse length by compression along scattering angle $\theta$. It is determined by the bandwidth of the chirped laser pulse $\Omega _L = (\tau _L^{-2} + \alpha ^2 \tau _L^2)^{1/2}$ and the angular dependent, double Doppler shift that results in an increase of the reflected frequency, as given by Eq. (1). The laser pulse can be compressed to very short pulses if a strong chirp is applied. In the following, when we refer to strong chirp, the rms frequency chirp $\alpha \tau _L$ is assumed to be much larger than the inherent pulse bandwidth given by the inverse of the laser pulse length $\tau _L^{-1}$. The bandwidth of a strongly chirped laser pulse is determined fully by the rms frequency chirp $\Omega _L \simeq \alpha \tau _L$.

Secondly, the term $\tau _\mathrm{CEM} = (1 + \cos \theta )/((1 + v/c ) \Omega _e)$ describes the shortest pulse that can be compressed along angle $\theta$ by the relativistic chirped mirror. Likewise, it is determined by the range of frequency components $\Omega _e = (\tau _e^{-2} + \eta ^2 c^4 \tau _e^2)^{1/2}$ that can be superradiantly reflected by the delay line of length $c\tau _e$ and a geometrical factor resulting from the projection of the electron density modulation on a particular scattering axis. If $\tau _\mathrm{CEM} > \tau _\mathrm{min}$, the mirror allows for partial reflection of the chirped laser pulse, such that full compression cannot be attained. In the ideal case, on the other hand, when $\tau _\mathrm{CEM} \ll \tau _\mathrm{min}$, the mirror allows for full compression and frequency up-conversion of the laser pulse.

The next contribution, $\tau _\perp = (\sigma _\perp /c) \sin \theta$, results from the transverse extent $\sigma _\perp$ of the mirror. Electrons that are separated transversely in space, emit radiation with a relative phase delay with respect to each other, leading to pulse broadening. However, when $\omega _1\tau _\perp > \pi$, destructive interference occurs, limiting the X-ray pulse energy, which we will address in the discussion section. Due to destructive interference, the transverse pulse broadening effect will then be restricted to about a single period.

The last term $\tau _\mathrm{GDD} = (\Gamma _1 - \Gamma _\mathrm{CEM})/(\tau _\mathrm{min}^2 + \tau _\mathrm{CEM}^2 + \tau _\perp ^2)^{1/2}$ is the pulse length due to (mis)matching of the spectral delay by the mirror to the laser frequency chirp. Here, $\Gamma _1 = \alpha \tau _L^2 \tau _\mathrm{min}^2$ is group delay dispersion (GDD) of the Doppler shifted laser pulse reflected by a single electron, which is a measure for the required spectral delay to reach $\tau _\mathrm{min}$. The term $\Gamma _\mathrm{CEM} = \eta c^2 \tau _e^2 \tau _\mathrm{CEM}^2$ is the GDD of the chirped electron mirror that quantifies its chromatic dispersion. A strongly chirped laser is optimally compressed by matching the two GDDs, which is true for head-on collision when the following condition is satisfied:

4 $$\begin{aligned} \alpha = \eta \left[ \frac{ c- v \cos \theta }{1 + \cos \theta }\right] ^2. \end{aligned}$$

Furthermore, superradiance is optimized by spectral overlap with the single electron radiation, which is achieved when the central bunching wavenumber $k_e$ is related to the central laser frequency $\omega _L$ by Eq. (2).

The shortest pulse is attained on-axis ($\tau _\perp = 0$) when the GDDs are matched ($\tau _\mathrm{GDD} = 0$). For strong chirp, the shortest pulse length for $\tau _\mathrm{min} = \tau _\mathrm{CEM}$ can be written as $\tau _{X} \simeq \sqrt{2}(4 \gamma ^2 \alpha \tau _L)^{-1}$ with $\gamma = (1-v^2/c^2)^{-1/2}$ the Lorentz factor. For instance, an 11 fs CEM with a kinetic energy of 5 MeV, scattering a matched, 5 ps laser pulse of central wavelength $\lambda _0 = 1000$ nm with a GDD of 0.033 ps$^2$, leads to a pulse length of $\tau _X = 20$ as at a wavelength of $\lambda _X \simeq \lambda _0/(4\gamma ^2) = 2$ nm. The uncompressed, single electron radiation pulse in this case is $\tau _1= \tau _\mathrm{min}(1 + \alpha ^2 \tau _L^4)^{1/2} = 11$ fs on-axis, three orders of magnitude longer than the compressed pulse.

**Figure 2:**
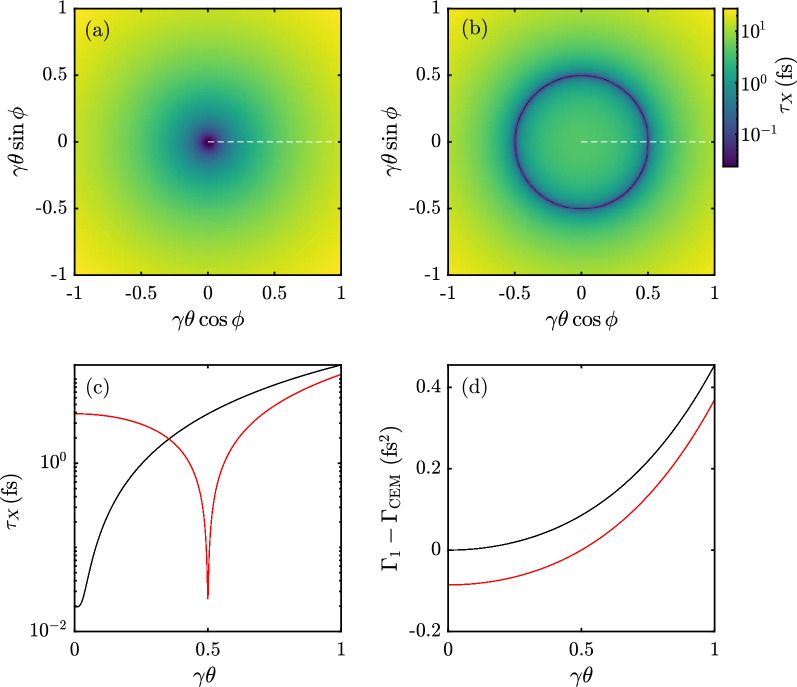
An 11 fs CEM irradiated by a GDD-matched 5 ps laser pulse. (**a**) Pulse length distribution for on axis matching. (**b**) Pulse length distribution for off axis matching. (**c**) Pulse length along white dashed line for on axis (black) and off axis (red) matching. (**d**) Difference in group delay dispersion between the superradiance induced by the mirror and the single electron radiation.

However, a mismatch in GDD leads to significant weaker compression of the pulse. Due to the angular dependence of the Doppler shift of TS, $\Gamma _{1}$ becomes increasingly larger going off-axis. The same holds for $\Gamma _\mathrm{CEM}$ which grows with $\theta$ due to the increase effective path length between radiation emitted from consecutive microbunches. The latter effect is much weaker, such that there can only be ideal matching along a single scattering direction. In addition to the inherently varying pulse length due to the relativistic Doppler shift, GDD (mis)matching results in an angular distribution of pulse lengths. In Fig. 2 the distribution of pulse lengths is shown on log scale for a infinitely thin ($\tau _\perp = 0$) mirror with matching axis along the central scattering axis (a) and along an off-axis angle (b) with the same electron beam energy and laser parameters as in the example of the previous paragraph. For the CEM in (a) the shortest pulse length (20 as) is found on-axis and becomes progressively longer off axis. The other mirror (b), matched off axis, reflects a radiation pulse at a slightly lower frequency. The shortest pulse length (24 as) is found at the matching angle where a ring of attosecond radiation is formed. The mismatch in GDD, therefore, is the dominant pulse broadening effect over red shifting due to the Doppler effect, which becomes even more clear in the projection given in Fig. 2c. Furthermore, the quantitative difference between group delay disperions $\Gamma _{1} - \Gamma _\mathrm{CEM}$ is shown in Fig. 2d.

**Figure 3:**
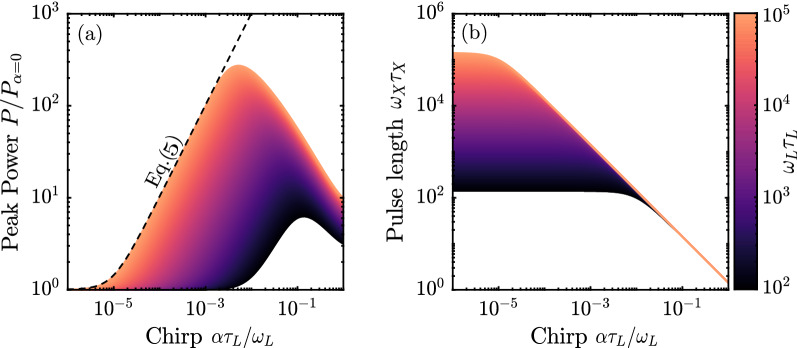
(**a**) Peak power *P* with respect to monochromatic peak power $P_{\alpha = 0}$ plotted against relative rms frequency chirp $\alpha \tau _L/\omega _L$ for an ideal on-axis matched CEM ($\sigma _\perp = 0$) as calculated analytically (dashed line) and by numerical integration of Eq. (28) for different laser pulse length (colors). The electron mirror is GDD matched with $\tau _\mathrm{CEM} = \tau _\mathrm{min}$. (**b**) Normalized pulse length $\omega _X \tau _X$ as function of relative rms frequency chirp for different laser pulse length (colors).

Pulse compression can greatly improve the peak power *P* of the generated X-rays. As shown in Eq. (27) the reflected pulse energy *W* for a thin mirror ($\tau _\perp \simeq 0$) consisting of $N_e$ electrons with matching along the central scattering axis is independent of the chirp rate. Therefore, we estimate that the peak power

5 $$\begin{aligned} P \simeq \frac{W}{\tau _X(0)} = \frac{3\pi \gamma ^2\sigma _T I_L\tau _L }{\omega _X\tau _X(0)} \frac{ b_1^2 N_e^2}{ \tau _e}, \end{aligned}$$

increases with chirp *i.e.* compression of the on-axis matched pulse length $\tau _X(0)$. Here, $\sigma _T$ is the Thomson cross-section, $I_L$ the peak intensity of the laser pulse, $b_1 \in [0,1]$ the quality of the electron beam density modulation and $\omega _X \simeq 4\gamma ^2\omega _L \simeq c k_e$. In Fig. 3a the peak power *P* reflected by a matched mirror with $\tau _\mathrm{CEM} = \tau _\mathrm{min}$ is plotted as function of the relative frequency chirp of the laser pulse $\alpha \tau _L/\omega _L$. In the figure, Eq. (5) (dashed line) for $\omega _L\tau _L = 10^5$ and *P*, as retrieved by numerical integration of the angular peak power distribution (see Eq. (28) in methods section), is plotted for different value of initial pulse length. The results in the figure confirm that the peak power increases by several orders of magnitude: For $\omega _L\tau _L = 10^5$ with chirp $\alpha \tau _L/\omega _L = 5\times 10^{-3}$ we find that the peak power $P = 275 P_{\alpha = 0}$ with $P_{\alpha = 0}$ the peak power of the unchirped case. Stronger chirps, however, counter-intuitively do not lead to further increase of peak power, although the pulse length linearly decreases as seen in Fig. 3b, leading to a mismatch between Eq. (5) and the numerical integrated data. This effect can be attributed to the angular dependence of GDD mismatching and the reflected energy distribution of the radiation: the pulse energy for an infinitely thin mirror is independent of chirp but is confined to an increasingly larger single electron scattering angle $\Theta _1\simeq \gamma ^{-1} \bar{\Omega }^{1/2}$, with $\bar{\Omega } = (\Omega _L^2/\omega _L^2 +\Omega _{e}^2/(ck_e)^2 )^{1/2}\simeq \sqrt{2} \alpha \tau _L/\omega _L$. Conversely, as the chirp rate grows, the angular width of the single electron radiation $\Theta _1$ increases, but the angular range in which the GDDs are matched $\Theta _\tau \simeq \gamma ^{-1}(2\Gamma _{1,\mathrm{CEM}})^{-1/2} (\tau _\mathrm{min}^2(0) + \tau _\mathrm{CEM}^2(0) )^{1/2} \simeq \gamma ^{-1}(\alpha \tau _L^2)^{-1/2}$ decreases. Therefore, at some chirp rate, part of the radiation starts to fall outside the matched angular range, and does not perfectly compress anymore. For a matched mirror with $\tau _\mathrm{CEM} = \tau _\mathrm{min}$ this happens when the chirp rate is equal to $\alpha _{P} = 2^{-1/4} (\omega _L/\tau _L^3)^{1/2}$. The peak power reaches its maximum value around around a slightly stronger chirp $\alpha _{P_\mathrm{max}} = 2 \alpha _{P}$. As such, Eq. (5) only holds for moderate chirps $\alpha < \alpha _P$. The dependency of the peak power on chirp rate, as shown in Fig. 3a, is independent of beam energy or reflected frequency.

To make previous results quantitative, we calculate the attosecond pulse properties generated by a CEM with bunching amplitude $b_1 = 0.5$, charge $Q = 7 \text { pC}$, pulse length $\tau _e = 100$ fs and beam energy $4.8 \text { MeV}$ ($\gamma = 10.4$) reflecting a $\tau _L = 12.5$ ps chirped laser pulse focused to a peak intensity of $I_L = 1.4 \times 10^{16} \text { W/cm}^2$ with a spectral distribution around central wavelength $\lambda _0 = 1 \mu \hbox {m}$. The mirror upconverts the laser pulse to the K-absorption edge of oxygen ($\lambda _X = 2.3 \text { nm}$) at the edge of the water window. We consider two cases of chirp: (i) high peak power chirp $\alpha = \alpha _{P_\mathrm{max}}$ corresponding to a GDD of 1.3 ps$^2$ and (ii) short pulse chirp $\alpha = 22\alpha _{P_\mathrm{max}}$ corresponding to a GDD of 0.06 ps$^2$, which can both be obtained using standard CPA optics. Different unchirped laser pulse lengths are needed (100 fs (i) and 5 fs (ii)) to meet the requirements on bandwidth for compression. For (i) we calculate that the reflected peak power is $P = 1.4 \text { GW}$ and pulse energy $W = 0.65 \mu \hbox {J}$, which is orders of magnitude higher than existing table-top sources in soft X-ray regime^16,33^. The pulse length of the high peak power case is $\tau _X = 253 \text { as}$. For the short pulse case (ii) the peak power is lower $P = 240 \text { MW}$ and $W = 0.39 \mu \hbox {J}$, but with a very short pulse length of $\tau _X = 12 \text { as}$ - corresponding to only 1.5 cycles - which has yet to be demonstrated experimentally in any kind of source^34–36^. In both cases, besides control over pulse length by the chirp, a constant phase offset can be applied to the laser pulse to change the carrier envelope phase (CEP) of the attosecond pulse, which is an important pulse characteristic for *e.g.* coherent imaging applications. Furthermore, the Thomson scattering process lends outstanding control over polarization, since the reflected radiation to a very high degree takes over the laser polarization^37^. The polarization state of the attosecond pulse can be chosen arbitrarily by the polarization of the driving laser, without altering the pulse energy or peak power of the pulse.

## Discussion

It is challenging to generate the intricate density modulation required for compression. At high electron beam energy, methods to produce chirped microbunching have been proposed^12^, and demonstrated^13^. Here, we mention two techniques that might be employed at the beam energy relevant to Thomson sources: First, by using conventional radio-frequency (RF) cavities, a density modulated electron bunch with zero bunching chirp can be non-linearly compressed to attain linearly chirped modulation^38^. The non-linearity may be obtained from non-linear compression fields or by non-linear velocity bunching at low beam energies. The second approach is similar to the method in ref.^13^, where a short magnetostatic undulator in combination with a co-propagating chirped laser pulse is used to impart a chirped energy modulation on the highly relativistic electron beam. The energy in the modulator is subsequently turned into a chirped density modulation using a magnetic chicane. At low beam energy, however, the magnetostatic undulator should be replaced by a short laser pulse to fulfill the resonance condition similar to Eq. (1) for optimal energy modulation. If the resonance condition is satisfied, a beat wave is formed travelling at the velocity of the electron bunch^39^. The ponderomotive force of the beat wave then imparts the energy modulation, which at the relevant beam energy turns into a density modulation by velocity bunching. The density modulation can be further compressed using RF-cavities with linear fields to attain bunching around the radiation frequency. However, this is not necessary if the bunching factor has a non zero component at X-ray frequency from a higher harmonic of the (local) bunching frequency. At harmonic *n* the chirp rate is increased to $n \eta$.

For the previous calculations we assumed that the electron mirror is cold, *i.e.* no spread in transverse or longitudinal momentum. However, in reality, an electron beam always has some finite transverse emittance and non-zero energy spread that influences the reflected X-ray pulses. Moreover, non-linear effects from strong laser fields, assumed negligible in the calculations, can potentially limit compression. All of the aforementioned effects induce broadening of the single electron radiation spectrum, which can be estimated from the resonance condition Eq. (1) by including a small general perturbation $\epsilon$ such that $\omega _1 \simeq 4\gamma ^2\omega _L /(1 + \epsilon )$. We estimate that perturbation $\epsilon$ does not hinder compression when the change in GDD matching satisfies the conditions $|\Gamma _1(\epsilon ) - \Gamma _\mathrm{CEM}| \le \tau _\mathrm{min}^2 + \tau _\mathrm{CEM}^2$, which for a strongly chirped laser compressed by a matched mirror is written as

6 $$\begin{aligned} \epsilon \le \frac{1}{2\alpha }\left[ \frac{1}{\tau _L^2} + \frac{1}{16\gamma ^4\tau _e^2}\right] = \gamma ^2\Theta _\tau ^2, \end{aligned}$$

where $\Theta _\tau$ is again the angular range of compression. Using Eqs. (1) and (9) we estimate that the effect of non-ideal conditions in general is given by $\epsilon \simeq \gamma ^2\Theta _e^2 + 2\delta \gamma /\gamma + A_0^2/2$ with $\Theta _e$ the angular spread of the electron beam, $\delta \gamma$ the energy spread and $A_0$ the normalized vector potential of the laser pulse. Quantum diffusion was not taken into account here since it requires an involved model describing superradiantly enhanced recoil, which is beyond the scope of this work.

**Figure 4:**
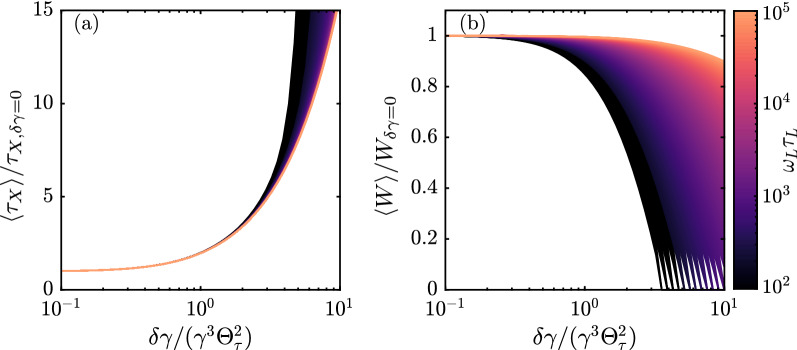
(**a**) Pulse length as function of energy spread for $\alpha = \alpha _{P_\mathrm{max}}$. The electron mirror is GDD matched with $\tau _\mathrm{CEM} = \tau _\mathrm{min}$. (**b**) Reflected pulse energy as function of energy spread for the same parameters.

The effect of energy spread on reflected pulse length and pulse energy is investigated numerically to check Eq. (6). We apply the weighted average $\langle x \rangle = (2\pi \delta \gamma ^2)^{-1/2} \int x\, \exp [\Delta \gamma ^2/(2\delta \gamma ^2)] d(\Delta \gamma )$ to the pulse length given by Eq. (3) and to the angular energy distribution given by Eq. (26), which we subsequently integrate over solid angle to find the pulse energy. The integration variable is given by $\Delta \gamma = \gamma ' - \gamma$ with $\gamma$ the average, on-resonance Lorentz factor. The results given in Fig. 4a confirm the estimated effect: if the energy spread $\delta \gamma /\gamma \ll \gamma ^2\Theta _\tau ^2$ the pulse length remains unaffected. In this regime, also the pulse energy is not reduced by energy spread as shown in Fig. 4b. Around the critical energy spread $\delta \gamma /\gamma \simeq \gamma ^2\Theta _\tau ^2/2$, the compression and pulse energy begins to be restricted by energy spread. When $\delta \gamma /\gamma \gg \gamma ^2\Theta _\tau ^2$, the pulse length is significantly longer that of the pulse reflected by the cold mirror. Besides slight variations in slope after the critical energy spread, the effect of energy spread on the pulse length is independent of initial matched pulse length. The reflected energy in this regime, for chirp $\alpha _{P_\mathrm{max}}$, is significantly reduced for large energy spread, and depends strongly on initial the pulse length.

Using Eq. (6) we find that for the high peak power case (i) in the previous section the critical normalized emittance $\epsilon _n \simeq \gamma \Theta _e \sigma _\perp$ at which the compression starts to be hindered is about 340 nm-rad (assuming an electron beam waist size of $\sigma _\perp = 5$ $\mu$m) and the critical energy spread is 12 keV. Such electron beam properties with bunch charge of tens of pC can be attained using a photoinjector^40^. For few cycle pulse generation the requirements are much more stringent: $\epsilon _n\le 80$ nm-rad for the same mirror waist size and $\delta \gamma m_ec^2 \le 0.5$ keV. These beam parameters, however, can still be achieved for bunch charges of several pC using a state-of-the-art thermionic gun^41^. Furthermore, we find that laser field strength proposed for the high peak power case (i) approximately corresponds to the critical value for $A_0 \le 0.1$. The compression to the near-single-cycle pulse (ii), for which the same field strength was proposed, will therefore be hindered significantly by ponderomotive broadening ($A_0 \le 0.02$). This can be compensated for, without lowering the peak intensity, by using a laser pulse with a smaller change in laser pulse envelope during superradiant reflection.

To sustain sufficient peak intensity over the interaction length, the laser Rayleigh length should be at least half the laser pulse length $c\tau _L$. To satisfy this condition for the proposed parameters in the examples, the pulse energy needed is 1.6 J. Nowadays, laser pulses with tens to hundreds of Joule of pulse energy are generated at several laser facilities around the world^42^. However, this has the disadvantage of requiring access to a laser facility. Furthermore, producing high energy laser pulses with sufficient bandwidth for compression to near-single cycle remains a challenge^43^. One way to circumvent the need for such high pulse energy, without having to admit significant reduction in peak power, is by focusing the chirped laser pulse using a lens with chromatic aberration. This enables a small laser waist for over 100 times the Rayleigh length and control over the speed at which the focus co- or counter-propagate along the propagation axis^44,45^. The so-called flying focus can be adjusted to the electron beam so that the required pulse energy is reduced by a factor $\sim 200$^46^. Such a focussing system potentially reduces the required pulse energy to the mJ level, which can be generated using a standard, compact laser system. Moreover, broadband mJ pulses at kHz repetition rate have been produced that can be compressed to near-single-cycle pulses^47^.

Another reason for considerable loss of attosecond pulse energy and consequently peak power is the transverse size of the mirror. Two regimes can be distinguished: In the pencil beam regime, where $\Theta _1\ll \Theta _\mathrm{CEM}$, with $\Theta _\mathrm{CEM} \simeq c(\omega _X\sigma _\perp )^{-1}$ the angle of superradiant emission, all reflected radiation is coherently amplified. In the beer-can beam regime, where $\Theta _1\gg \Theta _\mathrm{CEM}$, destructive interference due to the transverse size dominates, significantly limiting the pulse energy of the attosecond pulse. This effect can be compensated for by focusing to a small waist or going to high beam energy and introducing a oblique scattering geometry such that the angles better overlap^29^.

Besides the superradiant radiation that results in the compressed pulse, the electrons independently also emit incoherent radiation. Within relative bandwidth $\bar{\Omega }$, the incoherent energy reflected by the mirror is approximately given by $W_\mathrm{inc} = 3\pi \gamma ^2\sigma _T I_L \tau _L N_e \bar{\Omega }$. By definition the phase of the incoherently scattered radiation is random, and therefore does not interfere constructively, leading to a much longer pulse length $\tau _{1} = \tau _\mathrm{min}(1 + \alpha ^2\tau _L^4)^{1/2}$. The signal-to-noise ratio (SNR) in radiated power is approximately given by the ratio between incoherent and superradiant peak power:

7 $$\begin{aligned} \text {SNR} \simeq b_1^2 N_e \frac{\tau _\mathrm{min} \tau _\mathrm{CEM}}{\tau _\mathrm{min}^2 + \tau _\mathrm{CEM}^2} \frac{\tau _{1}}{\tau _e} \end{aligned}$$

where we assumed the pencil beam regime and that chirp rate $\alpha <\alpha _P$. Simplifying further to a matched mirror with $\tau _\mathrm{CEM} = \tau _\mathrm{min}$, the signal-to-noise ratio is simply given by $\text {SNR} \simeq 2^{-1/2}b_1^2N_e$. The superradiant emission dominates over the incoherent radiation if the bunch charge $N_e\gg b_1^{-2}$.

Summarizing, we propose a method to generate attosecond pulses using a long density modulated electron bunch that compresses and, simultaneously, frequency up-converts a chirped laser pulse. We have analytically calculated the generated angular pulse length distribution for arbitrary linear chirp, which we used to determine the optimal chirp parameters for compression of the laser pulse. Due to the angular distribution we found that the peak power is optimal for a relatively weak chirp rate $\alpha =\alpha _{P_\mathrm{max}}$. Furthermore, we estimate at which transverse emittance and energy spread compression is significantly affected. These findings can have great impact for the development of compact powerful soft X-ray sources.

## Methods

### Electron dynamics in a chirped laser pulse

We describe the interaction between an electron and a laser pulse in the framework of covariant electrodynamics, using the metric $g^{\mu \nu } = \mathrm{{diag}}(1,-1,-1,-1)$. In this classical description we neglect electron recoil, restricting the initial laser frequency $\omega _L$ to fulfill the condition $\gamma \hslash \omega _L/(m_e c^2) \ll 1$, where $\gamma _0$ is the Lorentz factor of the electron prior to interaction, $\hslash$ the reduced Planck’s constant, *c* the speed of light and $m_e$ the electron mass. Furthermore, we restrict our discussion to the linear regime where the vector potential amplitude of the laser pulse *A* satisfies $e A/m_e c \ll 1$, with *e* the elementary charge. In the linear regime the transverse momentum induced by the laser remains non-relativistic, which is desired in most experimental conditions to avoid spectral broadening^48^. From here, unless stated otherwise, we will normalize the relevant parameters as follows: charge is measured in units *e*, mass in units $m_e$, time is measured in the inverse of the initial laser frequency $\omega _L^{-1}$, length in $k_0^{-1} = c (\omega _L)^{-1}$ and (consequently) velocity is measured in units of *c* .

The four-potential of a chirped laser pulse can be written as:

8 $$\begin{aligned} A^{\mu }(\varphi ) = A_0 \exp \left[ -\frac{\varphi ^2}{2 \bar{\tau }_L^2}-i\left( \varphi + \frac{1}{2}\bar{\alpha }\varphi ^2\right) \right] \epsilon ^\mu \end{aligned}$$

where $A_0$ is the maximum vector potential amplitude, $\varphi = k_0^\nu x_\nu$ the optical phase with $k_0^\mu = (1,\mathbf {n}_0)$ the four wavevector of the laser pulse, $\mathbf {n}_0$ its propagation direction, $x^\mu = (t,\mathbf {x})$ the four-position, $\bar{\tau }_L$ the pulse length, $\bar{\alpha }$ the chirp rate and $\epsilon ^\mu = (0,\epsilon )$ the four-polarization. If the Lorentz gauge condition $\partial ^\nu A_\nu = 0$ holds and the vector potential only depends on $\varphi$, the exact solution to the Lorentz force equation is written as^49^

9 $$\begin{aligned} u^\mu (\tau ) = u_0^\mu + A^\mu (\varphi ) - \frac{1}{u_0^v k_{0v}}\left[ u_0^v A_v(\varphi )+ \frac{1}{2}A^\nu A_\nu (\varphi ) \right] k_0^\mu , \end{aligned}$$

where $u_0^\mu = (\gamma _0,\gamma _0 \mathbf {v}_0)$ is the initial four-velocity. The second term here describes the quiver momentum induced by the laser pulse. The terms within brackets describe the coupling with longitudinally oriented fields and momentum resulting from the ponderomotive force respectively. Since the maximum amplitude of the four-potential $A_0 \ll 1$, the second order ponderomotive term is considerably smaller than the others, such that it can be neglected in the following. For sake of clarity, we choose the laser polarization perpendicular to the initial velocity, making the last term zero. This simplification does not deprive our model of any useful physics, but it makes the expressions much easier to compute. Using that $d\varphi /d\tau = k_0^\nu u_\nu = k_0^\nu u_{0\nu }$ is a constant of motion, we can integrate to find the four-position

10 $$\begin{aligned} x^\mu (\varphi ) \simeq x_0^\mu + \frac{ u_0^\mu \varphi }{u_0^\nu k_{0\nu }} + \frac{i A^\mu (\varphi )}{\bar{\omega }(\varphi ) u_0^\nu k_{0\nu }}. \end{aligned}$$

Here, $x_0^\mu$ is the initial four-position of the electron with respect to the laser phase and instantaneous frequency $\bar{\omega }(\varphi ) = d\Phi /d\varphi$ with $\Phi$ the laser phase given by the imaginary part of the argument in Eq. (8). The second term describes the uniform motion by the initial velocity of the electron. The last accounts for the quiver motion with an amplitude that is proportional to the time the electron propagates through an optical cycle. It is important to note here that $x^\mu$ depends on $\varphi$ and therefore it is a recursive relation, which we must readdress for a correct description of coherent emission from the mirror. Equation (10) describes the electron motion correctly if the following conditions hold: $\chi _1 = |\bar{\alpha } \bar{\omega }^{-2}| \ll 1$ such that there is a notion of a pseudo period $T(\varphi ) = 2\pi / \bar{\omega }(\varphi )$, and $\chi _2 = |df/d\varphi (f \bar{\omega } )^{-1}|\ll 1$, so that the envelope of the vector potential amplitude $f(\varphi ) = \exp [-\varphi ^2/(2\bar{\tau }_L^2)]$ experiences almost no change over a pseudo period. Note that the last term of Eq. (10) diverge at $\varphi _c$, where $\bar{\omega }(\varphi _c)= 0$. However, since $\bar{\alpha } \ll \bar{\tau }_L^{-1}$ holds for a propagating pulse, the divergent terms have no physical meaning in the calculation of the scattered radiation discussed in the next part.

### Single electron four-potential

The radiation by a single electron is described by the solution to the inhomogeneous wave equation. In the far-field, where the scattering four-vector $k^\mu = (\omega ,\mathbf {k})$ is constant, the time-spectral solution to the wave equation is given by $A^\mu _{1}(\omega ,\mathbf {x}) = L_0 (x) \tilde{j}_1^\mu + \mathcal {O}(x^{-2})$ where $\tilde{j}_1^\mu = - (2\pi )^{-1}\int u^\mu (\tau )\exp (i k^\nu x_\nu ) d\tau$ is the spectral four-current of a single electron and $L_0(x) = (r_e/x) \exp (i \mathbf {k}\cdot \mathbf {x})$ is a quasi-invariant that describes the amplitude and phase of a spherical wave at a observer position *x* far away from the scattering charged particle and $r_e$ is the classical electron radius. Substitution of Eqs. (10) and (9) and linearizing the exponent in terms of $A_0$ gives

11 $$\begin{aligned} A^\mu _{1}(\omega ,\mathbf {x}) = - L_0(x) \exp (i k^\nu x_{0,\nu } ) \left[ \epsilon ^\mu - u_0^\mu \frac{k^\nu \epsilon _\nu }{ u_0^\kappa k_{\kappa }} \right] \tilde{A}(\omega ). \end{aligned}$$

Here we also expanded the oscillatory term $\bar{\omega }(\varphi )$ of (10) around the stationary phase $\varphi _s = (\omega /\omega _1-1)/\bar{\alpha }$, with $\omega /\omega _1 = k^\mu u_{0\mu }/(k_0^\nu u_{0\nu })$, of the integral that defines the resonance function $\tilde{A}(k) = (2 \pi k_0^\nu u_{0\nu })^{-1} \int A(\varphi ) \exp [i \omega \varphi /\omega _X ] d\varphi$ with $A(\varphi )$ the scalar part of Eq. (8). Integrating, while keeping only positive frequencies, results in:

12 $$\begin{aligned} \tilde{A}(\omega ) = \frac{A_0}{2\sqrt{2\pi } k_0^\nu u_{0\nu }} \frac{\bar{\tau }_L}{(1+\bar{\alpha }^2\bar{\tau }_L^4)^{1/4}} \exp \left[ - \frac{1}{2}\left( \frac{\omega }{\omega _1}-1\right) ^2\left( \frac{1}{\bar{\Omega }_1^2} - i\bar{\Gamma }_1\right) + i\Phi _1 \right] \end{aligned}$$

where $\bar{\Omega }_1 = (\bar{\tau }_L^{-2}+\bar{\alpha }^2 \bar{\tau }_L^2)^{1/2}$ is the rms relative bandwidth of the single electron vector potential, $\bar{\Gamma }_1 = \bar{\alpha }\bar{\tau }_L^2\bar{\Omega }_1^{-2}$ the normalized group delay dispersion and $\Phi _1 = \frac{1}{2}\arctan \bar{\alpha } \bar{\tau }_L^2$ a constant phase factor. The vector potential and resulting fields take over the relative frequency modulation of the laser pulse.

### Mirror four-potential and pulse length

The chirped electron mirror consist of $N_e$ electrons with initial position of the *j*th electron given by $x^\mu _{0,j}$. Using the superposition principle we can write

13 $$\begin{aligned} A^\mu _\mathrm{CEM}(\omega ,\mathbf {x}) = \sum _{j=1}^{N_e} A^\mu _{1,j}(\omega ,\mathbf {x}) = A^\mu _{1} \sum _{j=1}^{N_e} \exp \left[ ik^\nu x_{0,j\nu } \right] , \end{aligned}$$

where sum over phase factors is the form factor of the electrons which for $N_e\gg 1$ can be written in its continuous form:

14 $$\begin{aligned} b(\omega ) = \frac{1}{N_e}\sum _{j=1}^{N_e} \exp \left[ i k^\nu x_{0,j\nu } \right] \rightarrow \int _{-\infty }^\infty p^0 F(x,p) \exp \left[ i\left( k^\nu - \frac{\omega }{\omega _X}k_0^\nu \right) x_{\nu }\right] d^4pd^4x \end{aligned}$$

where *F*(*x*, *p*) is the initial 8-dimensional phase space distribution of the mirror prior to interaction with the driving laser pulse. The change in wave vector here is inferred by rewriting Eq. (10) for $A^\mu = 0$ to its non-recursive expression. Writing the discrete distribution of electrons as continuous takes away the incoherent contribution to the radiation, which is subdominant over the superradiant part when $b(\omega )\gg N_e^{-1/2}$. In the following we assume that all the electrons have the same initial velocity, *i.e.* the cold beam approximation (CBA). The distribution of a CEM in the CBA is given by

15 $$\begin{aligned} F(x,p) = \delta ^4(p^\mu -p_0^\mu ) \delta (x^0) \frac{\rho (\mathbf {x})}{\gamma Q} \end{aligned}$$

where $\rho (\mathbf {x})$ the charge density distribution and *Q* the total charge of the electron bunch. For normally distributed electrons with linear frequency modulated microbunches along its propagation (*z*-)direction, $\rho (\mathbf {x})$ is

16 $$\begin{aligned} \rho (\mathbf {x}) = \rho _0 \exp \left[ -\frac{1}{2}\mathbf {x}^T{S}^{-1}\mathbf {x}\right] \left( 1+ \Delta \cos \left[ k_e z + \frac{\bar{\eta } }{2} k_e^2 z^2 \right] \right) , \end{aligned}$$

where $S = \mathrm{diag}(\sigma _\perp ^{2},\sigma _\perp ^{2},\sigma _\parallel ^{2})$ with $\sigma _\perp$ the rms transverse CEM waist size, $\sigma _\parallel$ the rms mirror length, $\Delta$ the modulation depth, $k_e$ the bunching wavenumber and $\bar{\eta } = \eta /k_e^2$ the normalized microbunching chirp rate. The normalization constant that ensures $\int \rho d^3x = Q$ is given by

17 $$\begin{aligned} \rho _0 = \frac{Q}{(2\pi )^{3/2} \sigma _\perp ^2\sigma _\parallel } \left( 1 + \frac{\Delta }{(1 + \bar{\eta }^2 \bar{\tau }_e^4)^{1/4} } \exp \left[ -\frac{1}{2}\bar{\Omega }_e^{-2} \right] \cos \left[ \frac{1}{2}\bar{\Gamma }_e - \Phi _e \right] \right) ^{-1} \end{aligned}$$

where $\bar{\tau }_e = k_e \sigma _\parallel$, $\bar{\Omega }_e = (\bar{\tau }_e^{-2}+\bar{\eta } ^2\bar{\tau }_e^2)^{1/2}$ the rms relative bandwidth and $\bar{\Gamma }_e = \bar{\eta } \bar{\tau }_e^2\bar{\Omega }_e^{-2}$ the normalized group delay dispersion and $\Phi = \frac{1}{2}\arctan \bar{\alpha } \bar{\tau }_e$ a phase factor of the superradiant emission by the mirror. If $\bar{\Omega }_e \gg 1$, we can neglect the second term within the large round brackets such that $\rho _0 \simeq Q (2\pi )^{-3/2} \sigma _\perp ^{-2} \sigma _\parallel ^{-1}$. By combining Eqs. (14-17) the bunching factor for positive frequencies (and leaving the zero-band frequency out):

18 $$\begin{aligned} b(\omega ) = \frac{b_1}{(1 + \bar{\eta }^2 \bar{\tau }_e^4)^{1/4} } \exp \left[ - \frac{1}{2}\left( \frac{\omega }{\omega _\mathrm{CEM}}-1\right) ^2\left( \frac{1}{\bar{\Omega }_e^2} + i\bar{\Gamma }_e\right) + i\Phi _e - \frac{1}{2}\omega ^2\tau _\perp ^2 \right] \end{aligned}$$

where $\omega _\mathrm{CEM} = k_e (n_\parallel - n_{0,\parallel }/\omega _X )^{-1}$ the central resonant frequency of superradiance and $\tau _\perp = \sigma _\perp |\mathbf {n}_\perp - \mathbf {n}_{0,\perp }/\omega _X |$ the rms time delay between radiation from transversely separated electrons in the mirror with $\mathbf {n} = (\mathbf {n}_\perp ,n_\parallel )$ and $\mathbf {n}_0 = (\mathbf {n}_{0,\perp },n_{0,\parallel })$. Similar to the single electron scattering, the superradiant emission takes over the relative spectral properties of the microbunching, however at some shifted frequency dependent on the scattering and laser propagation direction with respect to the propagation direction of the mirror. For significant superradiant emission the resonant frequency $\omega _\mathrm{CEM}$ should be equal to $\omega _1$. However, since both depend on the scattering and laser direction differently, the condition $\omega _\mathrm{CEM}(\theta _r) = \omega _1(\theta _r)$ holds only at a single resonant scattering angle $\theta _r$ as measured from the mean propagation axis of the mirror.

Combining Eqs. (11), (12) and (18), and dropping normalization, yields the following expression for the mirror four-potential:

19 $$\begin{aligned} A^\mu _\mathrm{CEM}(\omega ,\mathbf {x})= & {} - L_0(x) N_e \left[ \epsilon ^\mu - u_0^\mu \frac{k^\nu \epsilon _\nu }{ u_0^\kappa k_{\kappa }} \right] \tilde{A}(\omega ) b(\omega ). \end{aligned}$$

20 $$\begin{aligned} A^\mu _\mathrm{CEM}(\omega ,\mathbf {x})= & {} -\frac{e \mu _0}{8\sqrt{2} \pi ^{3/2} } \frac{ A_0b_1 N_e }{c k_0^\nu u_{0\nu }} \left( \frac{ \bar{\tau }_e }{ \bar{\tau }_l \bar{\Omega }_e \bar{\Omega }_l}\right) ^{1/2} \left[ \epsilon ^\mu - u_0^\mu \frac{k^\nu \epsilon _\nu }{ u_0^\kappa k_{\kappa } }\right] \frac{1}{x}\exp \left[ -g(\omega ,\mathbf {x})\right] \end{aligned}$$

where $g(\omega ,\mathbf {x}) = \sum _{n=0}^2 \frac{1}{2}c_n(\mathbf {x)}\omega ^n$ a polynomial with complex coefficients $c_n$ given by

21 $$\begin{aligned} c_0= & {} \omega _{\text {CEM}}^2 \left( \tau _\mathrm{CEM}^2+i \Gamma _\mathrm{CEM}\right) +\omega _1^2\left( \tau _\mathrm{min}^2-i \Gamma _1\right) + 2i(\Phi _1 - \Phi _e) \end{aligned}$$

22 $$\begin{aligned} c_1(\mathbf {x})= & {} 2\omega _{\text {CEM}} \left( -\tau _\mathrm{CEM}^2-i \Gamma _\mathrm{CEM}\right) + 2\omega _1 \left( -\tau _\mathrm{min}^2+i \Gamma _1\right) + 2i \frac{1}{c} \mathbf {n}\cdot \mathbf {x} \end{aligned}$$

23 $$\begin{aligned} c_2= & {} \tau _\mathrm{CEM}^2+\tau _\mathrm{min}^2+ \tau _{\perp }^2 + i(\Gamma _\mathrm{CEM} - \Gamma _1) \end{aligned}$$

where we introduced the quantities $\tau _\mathrm{CEM,min} = (\omega _\mathrm{CEM,1} \bar{\Omega }_{e ,l})^{-1}$ and $\Gamma _\mathrm{CEM,1} = \bar{\Gamma }_{e ,l} \omega _\mathrm{CEM,1}^{-2}$, being the inverse bandwidth and group delay dispersion of the mirror and single electron emission respectively. The rms reflected pulse length can be found directly from the $c_2$ term as follows

24 $$\begin{aligned} \tau _X = \left[ \frac{c_2 c_2^*}{c_2 + c_2^*} \right] ^{1/2} = \left[ \tau _\mathrm{CEM}^2+\tau _\mathrm{min}^2 + \tau _{\perp }^2 + \frac{(\Gamma _\mathrm{CEM} - \Gamma _1)^2}{\tau _\mathrm{CEM}^2+\tau _\mathrm{min}^2 + \tau _{\perp }^2}\right] ^{1/2}, \end{aligned}$$

which is smallest when the GDDs are matched $\Delta \Gamma = \Gamma _\mathrm{CEM} - \Gamma _1 = 0$ and $\tau _{\perp } = 0$, which in general does not have to be on axis.

### Distribution of reflected energy and peak power

The energy reflected per unit frequency per unit solid angle in dimensional form is given by^50^:

25 $$\begin{aligned} \frac{d^2 W}{d\omega d\vartheta } = 4\pi \epsilon _0 c\omega ^2 x^2 A^\nu _\mathrm{CEM} A^{*}_{\mathrm{CEM }\nu } = \frac{ \alpha _f \bar{\tau }_e \hslash \omega ^2 A_0^2 b_1^2 N_e^2 }{8\pi (k_0^\nu u_{0\nu })^2 \bar{\tau }_l \bar{\Omega }_e \bar{\Omega }_l} \left[ 1 - \left( \frac{c k^\nu \epsilon _\nu }{ u_0^\kappa k_{\kappa } }\right) ^2\right] \exp \left[ -f(\omega )\right] , \end{aligned}$$

where $d\vartheta =\sin \theta d\theta d\phi$ differential solid angle, $\alpha _f$ the fine structure constant and $f(\omega ) = \sum _{n=0}^2 r_n\omega ^n$ with $r_n = \frac{1}{2}( c_n(\mathbf {x}) + c^*_n(\mathbf {x}))$ the real part of the coefficients given by (21)–(23). The energy distribution can be integrated analytically over angular frequency to find the angular distribution of energy:

26 $$\begin{aligned} \frac{d W }{d\vartheta } = \frac{ \alpha _f \bar{\tau }_e \hslash \omega ^2 A_0^2 b_1^2 N_e^2 }{32\sqrt{\pi } (k_0^\nu u_{0\nu })^2 \bar{\tau }_l \bar{\Omega }_e \bar{\Omega }_l} \left[ 1 - \left( \frac{c k^\nu \epsilon _\nu }{ u_0^\kappa k_{\kappa } }\right) ^2\right] \frac{2r_2 + r_1^2}{r_2^{5/2}} \exp \left[ \frac{r_1^2}{4 r_2} - r_0 \right] . \end{aligned}$$

Equation (26) can be integrated over solid angle for small scattering angles. Assuming that a infinitely thin $\sigma _\perp \simeq 0$ mirror compresses a counterpropagating laser pulse with GDD matching along the central axis, we find that

27 $$\begin{aligned} W \simeq \frac{\pi }{4}\alpha _f A_0^2 b_1^2 N_e^2 \frac{\bar{\tau }_L}{\bar{\tau }_e}\hslash \omega _X, \end{aligned}$$

which is independent of chirp rate. Since we also know the pulse length distribution (24), reflected by the mirror we can calculate the angular peak power distribution, which is simply:

28 $$\begin{aligned} \frac{d P}{d\vartheta } = \frac{1}{\tau _X}\frac{d W }{d\vartheta }. \end{aligned}$$

Equation (28) can be integrated over solid angle to find the reflected peak power.

## Data Availability

All data generated or analysed during this study are included in this published article.
